# Optimizing perioperative treatment for potentially resectable stage III squamous cell lung carcinoma: promising results of a condensed four-cycle regimen with tislelizumaband chemotherapy

**DOI:** 10.1186/s12916-024-03462-4

**Published:** 2024-06-10

**Authors:** Jianzhen Shan, Zhen Liu, Songan Chen, Chengli Du, Bing Li, Lingxiang Ruan, Mei Kong, Lingjie Wang, Miaoyan Du, Shuo Shi, Guoliang Qiao, Tian Tian, Zhengliang Tu

**Affiliations:** 1 Department of Medical Oncology, The First Affiliated Hospital, Zhejiang University School of Medicine, No. 79 Qingchun Road, Hangzhou, Shangcheng District China; 2 Cancer Center, Zhejiang University, Hangzhou, 310058 Zhejiang China; 3 Department of Medicine, Burning Rock Biotech, Guangzhou, China; 4 Department of Thoracic Surgery, The First Affiliated Hospital, Zhejiang University School of Medicine, No. 79 Qingchun Road, Hangzhou, Shangcheng District China; 5 Department of Radiology, The First Affiliated Hospital, Zhejiang University School of Medicine, Hangzhou, China; 6 Department of Pathology, The First Affiliated Hospital, Zhejiang University School of Medicine, Hangzhou, China; 7 Data Science Department, Burning Rock Biotech, Guangzhou, China

**Keywords:** Perioperative therapy, Squamous cell lung carcinoma, Major pathologic response, Biomarkers, Tislelizumab

## Abstract

**Background:**

The standard care for resectable non-small cell lung cancer (NSCLC) involves perioperative therapy combining chemotherapy and immune checkpoint inhibitors, typically lasting 6 to 12 months. However, the optimal treatment strategies for potentially resectable squamous cell lung carcinoma (SCC) remain unclear. This Phase 2 trial aimed to assess the efficacy and safety of a condensed four-cycle perioperative treatment regimen with tislelizumab combined with chemotherapy in patients with potentially resectable stage III SCC.

**Methods:**

Patients with potentially resectable stage IIIA-IIIB (N2) SCC received intravenous tislelizumab, albumin-bound paclitaxel, and carboplatin for up to four cycles. The primary endpoints were major pathologic response (MPR) and incidence of treatment-related adverse events. Safety and potential biomarkers for efficacy prediction were also assessed.

**Results:**

Among 35 enrolled patients, 32 underwent surgery with R0 resection achieved in all cases. MPR was achieved in 24 patients and pathological complete response (pCR) in 14 patients. Radiographic objective response was observed in 31 patients. The 12-month and 24-month event-free survival rate was 85.7 and 61.0%, respectively. Four patients experienced grade 3 or 4 adverse events. Tumor tissue based next-generation sequencing revealed the potential associations between several biomarkers and pathological response, including tumor neoantigen burden score, 18-gene expression profile score, CD8 + T cells, M1/M2 macrophages ratio and interferon‐gamma expression level. Besides, circulating tumor DNA (ctDNA) dynamics and concentration were also associated with pathological response and the presence of ctDNA at postoperative month 1 was a strong predictor for disease relapse. Furthermore, metagenomic sequencing in bronchoalveolar lavage fluid demonstrated *Streptococcus* was the most abundant genus in the pCR group.

**Conclusions:**

A condensed four-cycle perioperative treatment regimen of tislelizumab combined with chemotherapy demonstrated promising efficacy and manageable toxicities in potentially resectable stage III SCC. Specific biomarkers showed potential for predicting treatment efficacy and the mechanism of superior antitumor response of pCR patients was preliminarily and indirectly explored.

**Trial registration:**

ClinicalTrials.gov, NCT05024266. Registered August 27, 2021.

**Supplementary Information:**

The online version contains supplementary material available at 10.1186/s12916-024-03462-4.

## Background

Stage III non-small cell lung cancer (NSCLC) is a locally advanced malignancy with adverse prognostic factors in the primary tumor and/or regional lymph node metastasis [1]. A considerable proportion of these patients have N2 disease, indicating mediastinal lymph node involvement, and are potentially resectable [2]. However, the outcomes after surgery remain unsatisfactory [3]. Recent studies have demonstrated the promising role of neoadjuvant therapy with programmed cell death protein 1 (PD-1) or programmed death-ligand 1 (PD-L1) inhibitors combined with chemotherapy in NSCLC, including stage III patients [4, 5]. The Keynote-671 trial [6] proposed a treatment regimen consisting of 4 cycles of neoadjuvant chemotherapy combined with immunotherapy, followed by up to 13 cycles of adjuvant immunotherapy. Similarly, the IMpower010 [7] and KEYNOTE-091 trials [8] provided evidences supporting the potential benefits of 1 year of adjuvant PD-1 therapy in NSCLC patients. On the other hand, the Checkmate 816 trial [9] explored the efficacy of 3 cycles of neoadjuvant chemotherapy combined with immunotherapy, with or without adjuvant chemotherapy or radiotherapy. Furthermore, the NADIM II trial [10] demonstrated that a regimen of 3 cycles of neoadjuvant chemotherapy combined with immunotherapy, followed by 6 months of adjuvant immunotherapy, could potentially benefit patients with NSCLC. Thus, the optimal perioperative therapy strategy for NSCLC patients remains controversial.

Squamous cell lung carcinoma (SCC) represents a major subtype of NSCLC, accounting for approximately 25% of all cases [11]. Distinguished from lung adenocarcinoma, SCC exhibits unique molecular characteristics and often presents as a central mass accompanied by lymph node metastasis, posing challenges for initial surgical interventions. However, previous studies investigating neoadjuvant therapies have predominantly focused on the overall NSCLC population, paying limited attention to SCC, particularly in stage III patients. Furthermore, the prolonged duration of treatment may be costly and increase the risks of immunotherapy-related adverse effects (AEs). As a result, there is an unmet need for innovative treatment strategies that can improve operability and decrease the risk of recurrence, specifically tailored to the unique requirements of SCC patients.

To address this issue, we conducted a phase II trial, known as the TACT trial, to evaluate the effectiveness and safety of a condensed perioperative treatment regimen comprising tislelizumab, albumin-bound paclitaxel, and carboplatin in patients with potentially resectable stage IIIA-IIIB (N2) SCC (clinicaltrials.gov identifier: NCT05024266). Previous studies have demonstrated the favorable efficacy and safety of tislelizumab in combination with nab-paclitaxel and carboplatin as a first-line therapy for SCC patients, with a hazard ratio of 0.478 (95% CI, 0.336–0.679) [12]. Our study aimed to assess the feasibility of combining traditional four-cycle chemotherapy with immunotherapy as the perioperative treatment strategy. Furthermore, we investigated potential biomarkers in tumor tissue, plasma, and bronchoalveolar lavage fluid (BALF) microbiota to identify patients who may derive benefits from this treatment regimen. Importantly, our study specifically focused on the perioperative period of potentially resectable stage III SCC and provides evidence that a condensed four-cycle perioperative chemotherapy and immunotherapy combination is promising for these patients.

## Methods

### Participants

This prospective, single-arm, phase II clinical trial was conducted at the First Affiliated Hospital, Zhejiang University School of Medicine. Patients aged between 18 and 70 years were eligible for inclusion in the study. The inclusion criteria were as follows: a histopathologically confirmed diagnosis of SCC at stage IIIA or IIIB (T3N2M0 and T4N2M0 only, the 8th edition of the American Joint Committee on Cancer staging system, we utilized clinical radiographic staging, primarily through enhanced CT scans, to assess nodal involvement, reserving PET-CT and invasive mediastinal staging (EBUS/EUS, mediastinoscopy) for cases where CT results were inconclusive), suitability for potentially complete surgical resection (R0), Eastern Cooperative Oncology Group (ECOG) performance status score of 0 or 1, presence of measurable disease, and adequate pulmonary and organ function. Patients were also required to provide fresh tumor samples. The exclusion criteria included multiple primary malignancies, active or history of autoimmune disease, active or suspected interstitial lung disease or moderate-to-severe pneumonia, human immunodeficiency virus or active hepatitis B or C virus infection, previous systemic antitumor therapy and chest radiation, and previous use of immunostimulants, immunosuppressants, and live vaccine within 4 weeks before the first dose of the study treatment.

### Treatment

Eligible patients were treated with intravenous tislelizumab (200 mg on day 1), albumin-bound paclitaxel (260 mg/m^2^ on day 1), and carboplatin (area under the curve, AUC, 5 on day 1), every 3 weeks for 2 cycles. Imaging evaluation was performed after two cycles, followed by multidisciplinary discussion of the feasibility of surgery. If deemed operable, the lesion was resected 22–40 days after the last treatment. If the lesion was still deemed inoperable but had shrunk, the original regimen was continued for one to two additional cycles. Postoperative chemotherapy continued with the original regimen for zero to two cycles, for a total of four cycles of perioperative immunotherapy plus chemotherapy. The use of long-acting G-CSF was both permitted and encouraged to maintain chemotherapy doses and ensure timely treatment, particularly following any instances of grade 3 neutropenia.

### Outcomes

The primary endpoints of this study were the major pathologic response (MPR) rate and incidence of treatment-related adverse events. The secondary endpoints included R0 resection rate, objective response rate (ORR), and event-free survival (EFS). The pathological response was determined through blinded independent pathologic review, following the recommendations of the International Association for the Study of Lung Cancer (IASLC) for the pathologic assessment of lung cancer resection specimens after neoadjuvant therapy [13]. MPR was defined as less than or equal to 10% viable tumor cells in the resection specimen, while pCR was defined as the absence of viable tumor cells in the resected specimen. ORR was determined by the investigator, with ORR defined as the proportion of patients with complete response (CR) or partial response (PR) according to Response Evaluation Criteria in Solid Tumors version 1.1 (RECIST v1.1). EFS was defined as the time from the first dose of the study drug to disease progression, local recurrence, distant metastasis, or death, whichever occurred first. Disease-free survival (DFS), defined as the time from surgical resection to local recurrence, distant metastasis, or death, whichever occurred first, was also calculated. Treatment-related AEs were monitored and recorded.

### Biomarker analysis

PD-L1 expression was evaluated using the PD-L1 IHC 22C3 pharmDx assay (Dako, Agilent Technologies, CA, USA) on tumor biopsies obtained prior to treatment. PD-L1 positivity was defined as a tumor proportion score (TPS) of ≥ 1%. Whole-exome sequencing (WES) was performed using the Twist Human Core Exome kit and NovaSeq 6000 sequencer, with data analysis carried out using the Illumina DRAGEN Bio-IT Platform. Total RNA was extracted from formalin-fixed paraffin-embedded samples and subjected to whole transcriptome sequencing (WTS) on the Novaseq 6000. Immune cell subset estimation, gene expression profile (GEP) scores of 18 T-cell-inflamed genes and expressions level of other immune-related genes were determined based on the WTS data. Circulating tumor DNA (ctDNA) analyses were conducted using a personalized ctDNA panel (PROPHET, Burning Rock Biotech, Guangzhou, China) consisting of 50 single-nucleotide variants derived from tumor WES [14]. HLA class I four-digit typing was determined using Optitype v1.3.3. Neoantigen prediction was conducted using NetMHCpan v4.0 and a peptide was considered a putative neoantigen if it exhibited a predicted binding affinity of < 500 nM. BALF was collected prior to neoadjuvant treatment. The microbial DNA was extracted, and the target genes were amplified and sequenced on a NovaSeq PE250 system. Additional details are provided in the Additional file 1.

### Statistical analysis

A total of 29 participants were required to achieve 80% power in detecting an MPR of 50% under a one-sided 2.5% alpha, assuming the null hypothesis of an MPR equals to 25%. Considering a 15% discontinuation rate, 35 patents were enrolled in the study. Efficacy and safety analyses were conducted on the intention-to-treat population, which included all patients regardless of whether they underwent surgery. MPR, pCR, and ORR were calculated using the Clopper-Pearson exact method. EFS and DFS were estimated using the Kaplan–Meier method, and the 95% CIs for median survival were calculated using the Clopper-Pearson exact method. Furthermore, the study explored the relationships between pathological response and biomarkers such as PD-L1 expression, tumor mutation burden (TMB), tumor neoantigen burden (TNB), gene expression signature in tumor tissues, ctDNA in peripheral blood, and microbiome in BALF. The *t* test was employed to assess the differences in the abundance of microbiome species between groups. All analyses used two-sided *P* values, and the significance level was set at 0.05 unless otherwise noted. SPSS software (version 26) and R software (version 4.1.2) were used for statistical analyses.

## Results

### Patients and treatment

Between July 2, 2021, and May 17, 2022, 56 patients with histopathologically confirmed SCC were screened for eligibility. Of these, 35 met the inclusion criteria and were enrolled in the study (Additional file 1: Fig. 1 and Additional file 2). All patients received neoadjuvant treatment consisting of two to four cycles of tislelizumab combined with chemotherapy (albumin-bound paclitaxel and carboplatin). Of the enrolled patients, 30 (85.7%) had stage IIIA disease and 5 (14.3%) had stage IIIB disease. Most of patients (34/35, 97.1%) had smoking history, and a significant proportion (25/35, 71.4%) had comorbidities such as hypertension, diabetes, and benign prostatic hyperplasia. These comorbidities were well controlled at the time of enrollment and graded as Common Terminology Criteria for Adverse Events (CTCAE) grade I. Detailed demographic and disease characteristics are presented in Table 1.

**Fig. 1:**
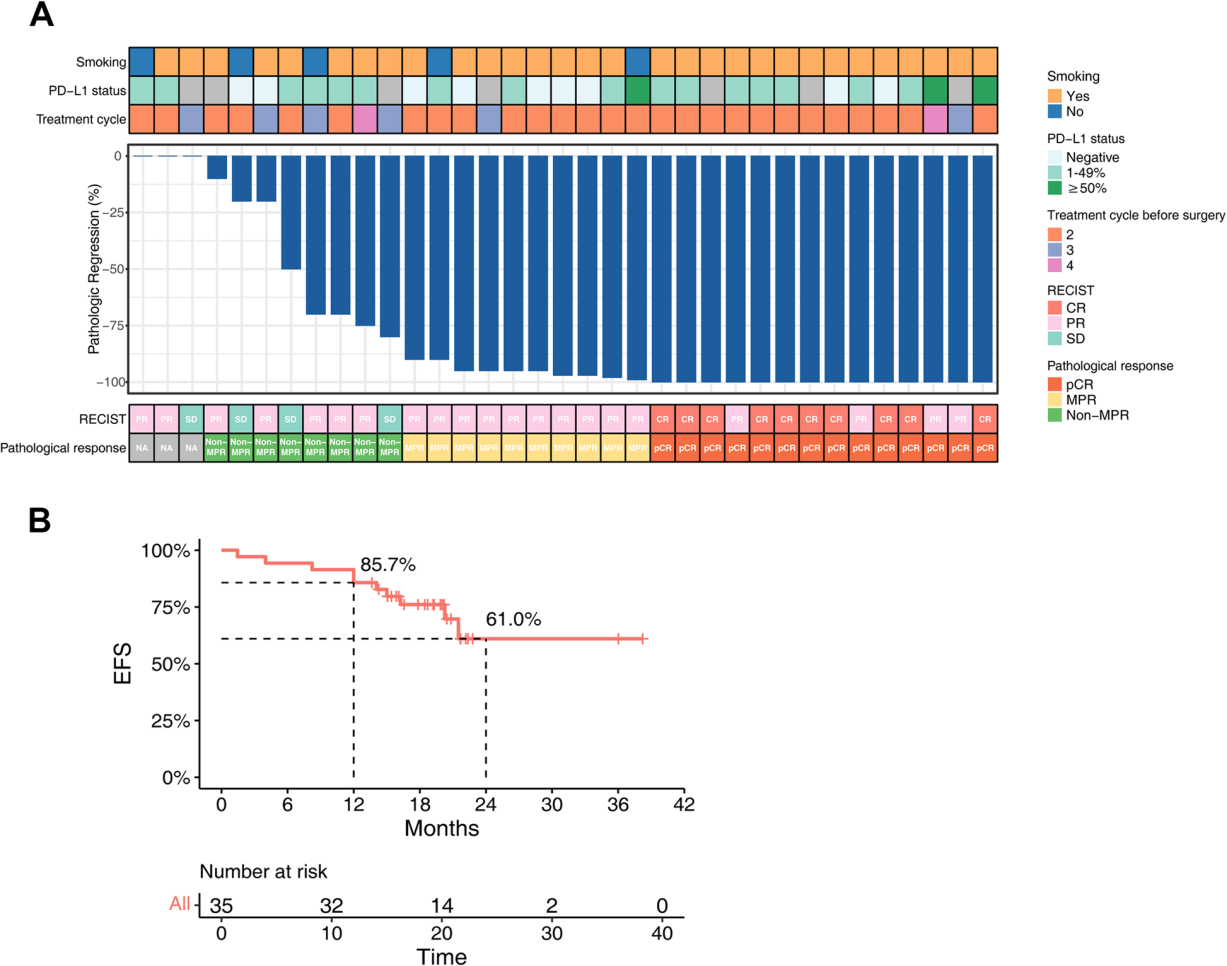
Overview of clinicopathological characteristics and clinical outcome of patients treated with neoadjuvant tislelizumab plus chemotherapy. **A** Pathologic regression and clinicopathological characteristics. **B** Kaplan–Meier survival curve for EFS

**Table 1:** Baseline demographic and clinical characteristics

Variables	Patients (*n* = 35)
**Age**	
Age, median (range), years	65 (48–70)
< 65, No. (%)	16 (45.7)
≥ 65, No. (%)	19 (54.2)
**Sex, *n* (%)**	
Male	35 (100)
**Clinical stage, *n* (%)**	
T1N2M0	7 (20)
T2N2M0	11 (31.4)
T3N1M0	8 (22.9)
T3N2M0	1 (2.9)
T4N0M0	2 (5.7)
T4N1M0	2 (5.7)
T4N2M0	4 (11.4)
**ECOG score, *n* (%)**	
0	16 (45.7)
1	19 (54.3)
**Smoking status, *n* (%)**	
Current or former	30 (85.7)
Never	5
**Treatment cycle before surgery, *n* (%)**	
2 cycles	27 (77.1)
3 cycles	6 (17.1)
4 cycles	2 (5.7)
**TPS PD-L1 (22C3), *n* (%)**	
< 1%	9 (25.7)
1–49%	16 (45.7)
≥ 50%	3 (8.6)
NA	7 (20)
**Comorbidities, *n* (%)**	
Yes	25 (71.4)
No	10 (28.6)

Of the 35 patients enrolled in the study, 32 (91.4%) underwent surgery and achieved R0 surgical resection. The surgical procedures performed included lobectomy in 21 patients (65.6%), sleeve lobectomy in 7 patients (21.9%), bilobectomy in 2 patients (6.3%), and pneumonectomy in 2 patients (6.3%) (Table 2). Notably, 24 out of 32 patients (75%) underwent minimally invasive surgery. Three patients did not undergo surgery: one patient had immunotherapy-induced hepatitis, whereas two patients opted not to undergo surgery for personal reasons.

**Table 2:** Surgical performance and pathological response

Outcomes	Patients (*n* = 32)
**R0 resection, *n* (%)**	32 (100)
**Surgical approach, *n* (%)**	
Thoracotomy	7 (21.9)
Minimally invasive	24 (75)
Minimally invasive to thoracotomy	1 (3.1)
**Resection type, *n* (%)**	
Sleeve lobectomy	7 (21.9)
Lobectomy	21 (65.6)
Bilobectomy	2 (6.3)
Pneumonectomy	2 (6.3)
**Pathological response, *n* (%)**	
pCR	14 (43.8)
MPR	24 (75)
Non-MPR	8 (25)
**T downstaging, *n* (%)**	27 (84.4)
T4 to T3	1 (3.1)
T4 to T1	3 (9.4)
T4 to T0	4 (12.5)
T3 to T1	5 (15.6)
T3 to T0	4 (12.5)
T2 to T1	7 (21.9)
T2 to T0	2 (6.3)
T1 to T0	1 (3.1)
**Nodal downstaging, *n* (%)**	**23 (71.9)**
N2 to N0	15 (46.9)
N2 to N1	3 (9.4)
N1 to N0	7 (21.9)

Among the 32 patients who underwent surgery, 30 patients received subsequent adjuvant therapy. Two patients did not receive adjuvant therapy and were under observation because they had already received neoadjuvant therapy for four cycles before surgery. One patient discontinued adjuvant therapy for personal reasons. Eventually, 29 patients completed adjuvant therapy. As of the cutoff date on July 6th, 2023, all patients had completed the treatment and were under follow-up.

### Efficacy

After 1–4 cycles of preoperative treatment with tislelizumab combined with chemotherapy, 31 of 35 patients (88.6%, 95% CI 77.5–99.7%) had an objective response, with 10 patients (28.6%) achieving CR, 21 patients (60%) achieving PR, and 4 patients (11.4%) achieving stable disease (SD). None of the patients had progressive disease during neoadjuvant therapy (Fig. 1A).

At the data cutoff, the median follow-up period was 16.7 months (95% CI 15.7–17.8). Out of the 35 patients, 9 (25.7%) experienced disease progression, disease occurrence, or death. The median EFS was not reached and the 12-month, 24-month EFS rate were 85.7% (95% CI 74.9–98.1%), 61.0% (95% CI 42.3–88.0%), respectively (Fig. 1B). All 3 patients (8.6%) who did not undergo surgery developed progressive disease, and 1 patient (2.9%) died. Among the 32 patients who underwent surgery, 5 (15.6%) had disease occurrence at the time of cutoff.

Of the 32 patients who underwent surgery, 24 (75%, 95% CI 59.1–90.9%) achieved MPR and 14 (43.8%, 95% CI 25.6–61.9%) achieved pCR (Fig. 1A and Table 2). Moreover, 27 out of 32 patients (84.4%, 95% CI 71.1–97.7%) achieved pathological T downstaging, and 23 patients (71.9%, 95% CI 55.4–88.3%) had nodal downstaging after neoadjuvant treatment. Notably, 15 patients (46.9%) had nodal downstaging from N2 to N0 (Table 2).

### Safety

All 35 patients experienced treatment-related AEs during the perioperative treatment (Table 3). The most common treatment-related AEs were hematologic events, including neutropenia (28 [80.0%] of 35 patients), anemia (19 [54.3%]), and thrombocytopenia (16 [45.7%]). The common nonhematologic adverse events were alopecia (25 [71.4%]), fatigue (6 [17.1%]), anorexia (5 [14.3%]), and myalgia (5 [14.3%]). Three patients (8.6%) experienced skin rash, which was evaluated as immune-related AEs. Four patients (11.4%) experienced AEs of grade 3 or 4, which were neutropenia (3 [8.6%] of 35 patients) and increased aspartate transaminase (AST) and alanine transaminase (ALT) (1 [2.9%]). All grade 3 and 4 AEs occurred before surgery. None grade 5 AEs occurred and none of the AEs led to treatment discontinuation or dose reduction.

**Table 3:** Treatment-related AEs

Adverse events	Any grade No. (%)	Grades 3–4 No. (%)
Hematologic adverse events		
Neutropenia	28 (80)	3 (8.6)
Anemia	19 (54.3)	0
Thrombocytopenia	16 (45.7)	0
Nonhematologic adverse events		
Alopecia	25 (71.4)	0
Fatigue	6 (17.1)	0
Anorexia	5 (14.3)	0
Myalgia	5 (14.3)	0
Paresthesia	4 (11.4)	0
Nausea	3 (8.6)	0
Rash	3 (8.6)	0
Constipation	2 (5.7)	0
Increased AST	2 (5.7)	1 (2.9)
Increased ALT	2 (5.7)	1 (2.9)
Increased blood creatinine	1 (2.9)	0
Increased bilirubin	1 (2.9)	0

*AST* aspartate transaminase, *ALT* alanine transaminase

### Analysis of biomarkers

**Fig. 2:**
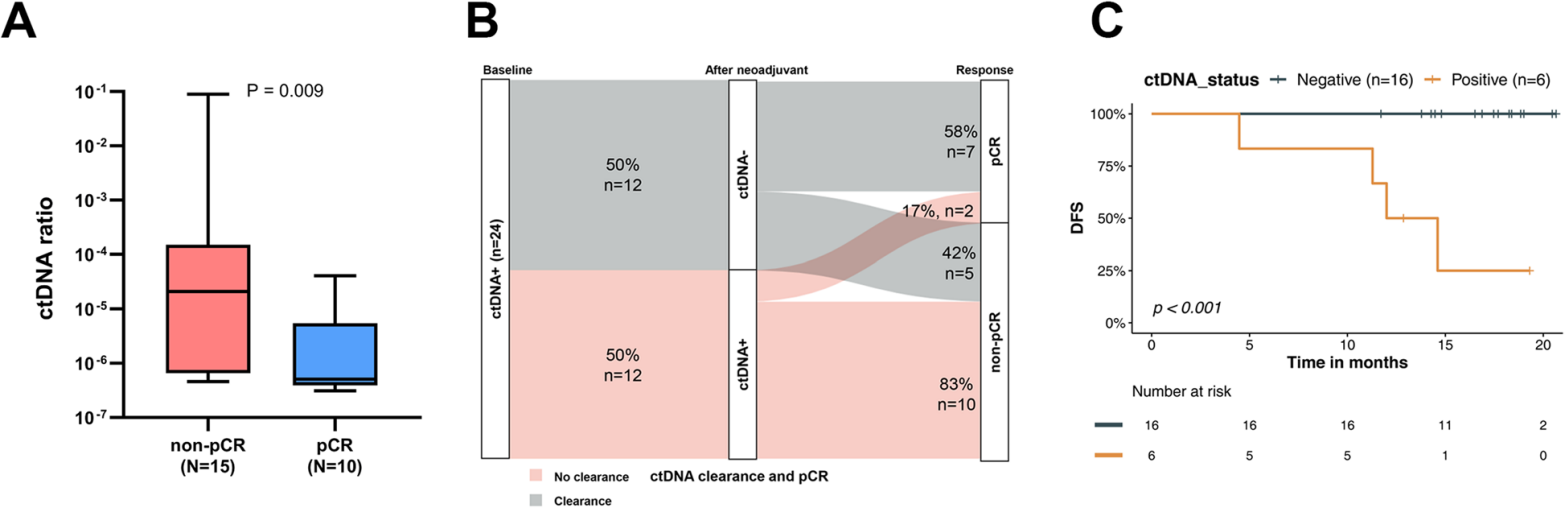
Association of ctDNA with pathological response and survival. **A** ctDNA concentration after neoadjuvant treatment between pCR and non-pCR. **B** Sankey plot showing ctDNA dynamics (clearance or non-clearance) versus response. **C** DFS of patients with ctDNA positive and ctDNA negative after surgery

Next-generation sequencing and PD-L1 expression analysis were conducted to explore potential biomarkers for predicting pathological response. Firstly, ctDNA concentration and dynamic changes at baseline and after neoadjuvant treatment were compared between pCR and non-pCR patients. At baseline, nearly all pCR (91%, 10/11) and non-pCR (100%, 15/15) patients tested positive for ctDNA. There was no significant difference in ctDNA content between the two groups (*P* = 0.134, Additional file 1: Fig. S2A). However, after neoadjuvant treatment, the positive rate of ctDNA in the pCR group decreased to 20.0% (2/10), compared to 66.7% (10/15) in the non-pCR group (*P* = 0.06, Additional file 1: Fig. S2B). Additionally, the pCR group exhibited a significantly lower ctDNA concentration (*P* = 0.0105, Fig. 2A). Out of the 24 patients who were ctDNA positive at baseline and had corresponding plasma measurements after neoadjuvant treatment, 12 remained ctDNA positive at that time point. Patients without ctDNA clearance tended to have residual disease compared to those with ctDNA clearance, although no statistically significant difference was observed, possibly due to the small sample size (*P* = 0.092, Fig. 2B). The positive predictive value (PPV) was 83% (10/12) while the negative predictive value (NPV) was 58% (7/12). Furthermore, we investigated the difference in EFS between the ctDNA clearance group and no clearance group. As expected, the ctDNA clearance group demonstrated a trend towards better survival (*P* = 0.196, Additional file 1: Fig. S2C). Additionally, we evaluated the prognostic significance of postoperative ctDNA. The Kaplan–Meier estimates indicated that the presence of ctDNA at postoperative 1 month postoperative was highly predictive of relapse (*P* < 0.001, Fig. 2C).

**Fig. 3:**
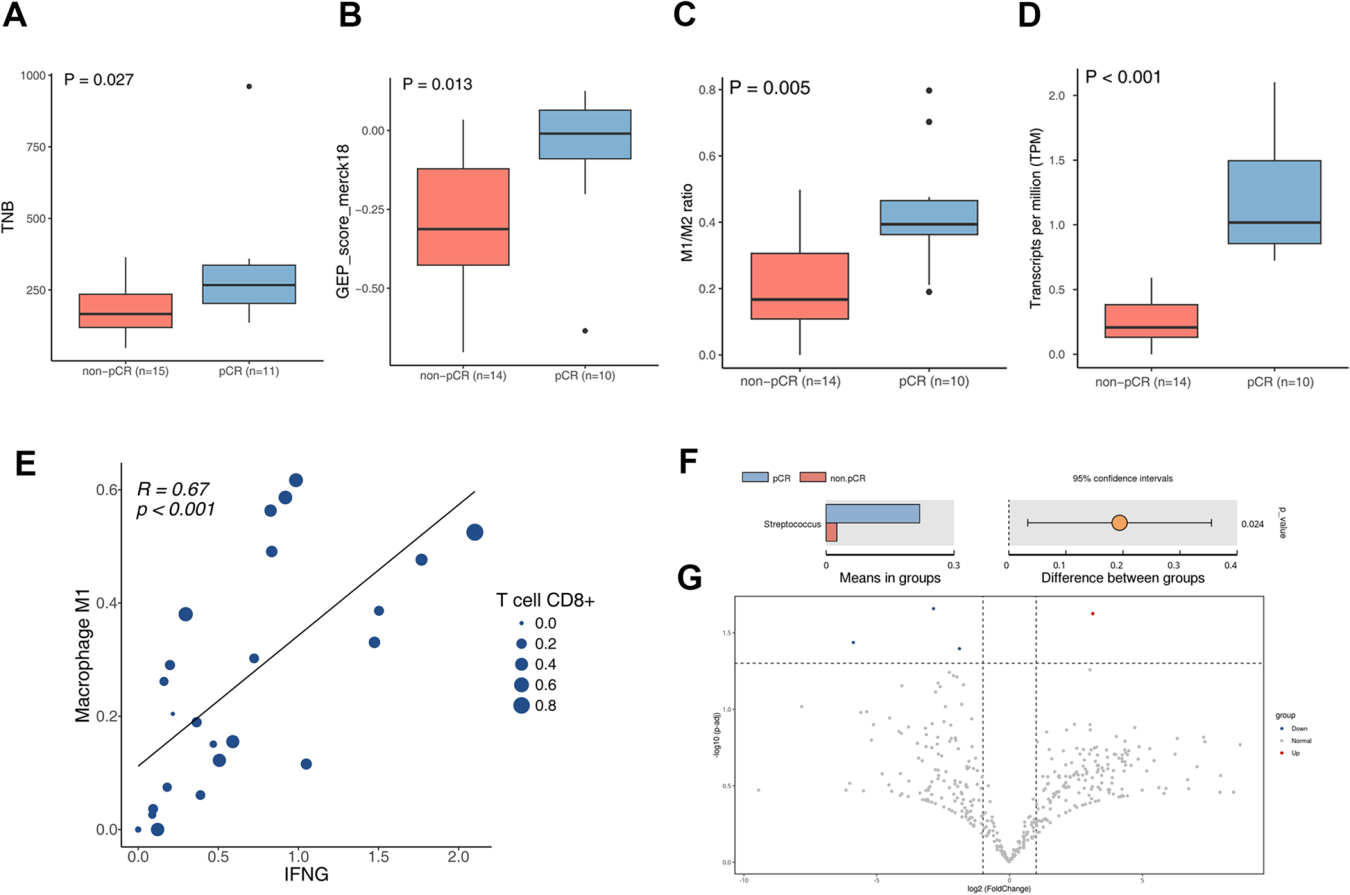
Potential biomarkers for predicting pathological response in baseline tumor tissue and BALF. **A** TNB score, (**B**) GEP 18-gene score, (**C**) the ratio of M1 macrophages to M2 macrophages, (**D**) IFNG expression level, (**E**) correlation between IFNG expression level, M1 Macrophages and CD8 + T cells, (**F**) bar chart and (**G**) volcano plot of intergroup differences in BALF microbial species between pCR group and non-pCR group

Based on the available WES (*n* = 28) and WTS (*n* = 26) data obtained from baseline tumor tissue, we have identified several biomarkers potentially associated with pathological response. Specifically, patients with pCR demonstrated significantly higher median TNB score (267 vs. 166, *P* = 0.027, Fig. 3A) and Merck 18-gene score (*P* = 0.013, Fig. 3B) compared to the non-pCR group. Moreover, the results of immune cell population estimation showed that CD8 + T cells and M1 macrophages, two classical immune cell populations with antitumor activity, were significantly more abundant in pCR patients than those in non-pCR patients (Additional file 1: Fig. S3A). While the M2 macrophages were also more abundant in pCR patients (Additional file 1: Fig. S3A), the ratio of M1/M2 macrophages in pCR patients was still significantly higher (*P* = 0.0045, Fig. 3C).

Interestingly, we also found that the expression level of *IFNG*, which encodes interferon-gamma (IFN-γ) crucial for antitumor responses, was significantly higher in pCR patients than that in non-pCR patients (*P* < 0.001, Fig. 3D), with an area under the curve (AUC) of 0.96 (Additional file 1: Fig. S3B). IFN-γ, produced by immune cells such as T cells and NK cells, plays a pivotal role in antitumor responses by stimulating macrophages. We further examined the potential correlation between the expression level of *IFNG* and the abundance of several immune cells. As expected, there was a significantly positive correlation between the *IFNG* expression level and the abundance of CD8 + T cells, as well as M1 macrophages (Fig. 3E, Additional file 1: Fig. S3C-D). Furthermore, patients with high IFNG expression appeared to have a tendency for better prognosis when using the upper quartile as a cutoff (Additional file 1: Fig. S3E). Meanwhile, TMB and PD-L1 TPS scores were also analyzed between the pCR and non-pCR groups. Although no statistically significant differences were found, there were tendencies of higher median TMB and PD-L1 TPS scores in pCR patients (Additional file 1: Fig. S3F-G).

In this study, we also explored the diversity of the microbiome in BALF prior to treatment to better understand the variations in microbial communities among patients who achieved pCR and those who did not. We successfully obtained BALF samples from 9 pCR patients and 14 non-pCR patients for metagenomic sequencing. Our analysis revealed *Streptococcus* as the most abundant bacterial genus in the pCR group, compared to the non-pCR groups (*P* = 0.024, Fig. 3F,G). This finding suggests a potential association between these bacteria and treatment response.

## Discussion

To our knowledge, this is the first prospective study to evaluate the efficacy and safety of neoadjuvant treatment with tislelizumab combined with chemotherapy in patients with potentially resectable stage IIIA-IIIB (N2) SCC. The neoadjuvant therapy did not increase surgical complexity, with 75% of patients undergoing minimally invasive surgery.

Several recent studies have assessed the efficacy and safety of immunotherapy combined with chemotherapy as neoadjuvant therapy in NSCLC. In the CheckMate 816 trial [9], patients with resectable stage IB to IIIA NSCLC received nivolumab combined with platinum-based chemotherapy and achieved a 60.9% MPR rate, 24% pCR rate and 76.1% 12-month EFS rate. Grade 3 or 4 AEs occurred in 33.5% of patients in the nivolumab combined with chemotherapy group. The NADIM trial [15] enrolled patients with stage IIIA NSCLC, who received paclitaxel and carboplatin combined with nivolumab for three cycles, followed by adjuvant nivolumab monotherapy for 1 year. The MPR rate was 83% and the pCR rate was 63.4%. Thirty percent of patients had treatment-related grade 3 or worse AEs. The subsequent NADIM II trial [16] enrolled stage IIIA/B NSCLC patients, the MPR rate was 52% and the pCR rate was 36%. In another trial, perioperative treatment with adebrelimab (a programmed death-ligand 1 antibody) combined with nab-paclitaxel and carboplatin was evaluated in resectable stage II to III NSCLC patients, and 51.4% of patients achieved MPR, while 29.7% of patients achieved pCR. Grade 3 or worse AEs were reported in 78.4% of patients [17]. Four recent perioperative trials for NSCLC, Checkmate 816 [9], AEGEAN [18], Neotorch [19], and KEYNOTE-671 also demonstrated that perioperative immunotherapy resulted in significantly longer EFS than chemotherapy alone. These trials reported 12-month EFS rates of 76.1, 73.4, 84.4, and 73.2% and 24-month EFS rates of 63.8, 63.3, 64.7, and 62.4%, respectively. In our trial, we observed a 100% R0 resection rate, 75% MPR rate, 43.8% pCR rate, 85.7% 12-month EFS rate, and 61.0% 24-month EFS rate in stage IIIA-IIIB (N2) SCC patients after four cycles of perioperative treatment. These results were comparable with the findings reported in many previous studies. Additionally, the majority of adverse events were grades 1–2, and only 11.4% of patients experienced grade 3–4 toxicities. The high R0 resection and MPR rates may improve surgical outcomes and ultimately extend survival.

It is worth noting that our trial employed a flexible and short-term treatment regimen. Surgical evaluation was conducted after 2 cycles of preoperative therapy, and if surgery was deemed infeasible, the original treatment was continued for an additional 1–2 cycles, for a total of 4 cycles of therapy. In contrast, other perioperative trials such as Checkmate 816 [9], NADIM [15], NADIM II [16], AEGEAN [18], Neotorch [19], and KEYNOTE-671 [6, 20] involved longer treatment durations, ranging from 3 to 19 cycles of immunotherapy. However, the beneficial effects of these regimens were often modest and associated with increased costs and drug-related toxicities. In our trial, we achieved comparable or MPR rate, pCR rate, and 12-month EFS rate with a shorter treatment duration, suggesting that a 4-cycle perioperative treatment with tislelizumab plus chemotherapy may be a more efficient and cost-effective approach for potentially resectable stage IIIA-IIIB SCC patients.

Our findings, in potentially resectable stage IIIA-IIIB (N2) SCC, demonstrating the prognostic utility of detectable ctDNA post-operatively and dramatic survival difference based on ctDNA clearance align with research from the IMpower010 trial in early-stage NSCLC. Using an ultra-sensitive ctDNA assay, they established post-surgical ctDNA detection as an independent predictor of shorter disease-free survival (HR 2.26, *p* < 0.001), while chemotherapy-induced ctDNA clearance predicted superior outcomes (HR 0.17, *p* < 0.001). Similarly, we found ctDNA dynamics after neoadjuvant therapy can be used to predict pathological response, with a high PPV (83%, 10/12) to predict non-pCR, which is consistent with the findings of the Checkmate 816 trial (100%, 19/19) [9]. What is more, our data revealed that patients with positive ctDNA at postoperative month 1 had significantly shorter DFS than those with negative ctDNA, which is consistent with numerous previous reports [21–24]. In these observational studies, only the positive ctDNA patients, other than those negative ctDNA patients, may benefit from adjuvant therapy. Yue et al. also demonstrated detectable ctDNA after neoadjuvant therapy trended to correlate with inferior recurrence-free survival in stage IB–IIIA NSCLC [25]. In view of the fact that the optimal adjuvant therapy strategies for patients who received neoadjuvant immunotherapy are not well established, ctDNA status after neoadjuvant therapy and after surgery may play an important role in guiding personalized adjuvant therapy. However, prospective intervention studies are needed.

We also investigated the potential biomarkers in baseline tumor tissues for predicting pathological response. Our study found that significantly higher TNB score were presented in the pCR group, which was deemed to produce neoantigens and generate an inflammatory microenvironment that ultimately led to improved outcomes following immunotherapy [26]. As expected, pCR patients had significantly greater enrichment of classical immune cell populations that mediate antitumor roles, such as CD8 + T cells, M1 macrophages. Additionally, the ratio of M1/M2 macrophages in non-pCR patients was significantly lower, indicating that the immune microenvironment of non-pCR patients was relatively inhibitory [27, 28]. Beyond that, we found the pCR group had a significantly higher GEP score than the non-pCR group (*P* = 0.011). To the best of our knowledge, this is the first study to demonstrate the predictive value of GEP-18-gene score in the neoadjuvant setting for potentially resectable IIIA-IIIB (N2) SCC. Our results also suggested that high levels of IFNG can be used as a powerful predictor to distinguish patients whose tumors will achieve pCR, which was consistent with the report from NADIM trial [29]. As we all known, IFN-γ is produced by immune cells such as T cells and NK cells and further activates macrophages to plays crucial role in antitumor responses, as well as upregulating antigen processing and presentation pathways [30]. Excitedly, we further confirmed a significantly positive correlation between the *IFNG* expression level and CD8 + T cells, as well as M1 macrophages. These results implied that another mechanism for the superior antitumor response of pCR patients via more CD8 + T cells assembled to produce more IFN-γ and made more M1 macrophages be stimulated. All of these findings indicated that pCR patients had a relatively active pre-established tumor immune microenvironment at baseline than non-pCR patients which resulted in a superior antitumor response.

The potential role of gut microbes in modulating immunity and antitumor responses in various cancers, including lung cancer, has been well recognized in previous studies [31, 32]. However, the microbiome in BALF, which is closely related to the lung cancer microenvironment, has received little attention. Masuhiro et al. showed that checkpoint inhibitor responders had a greater diversity of the lung microbiome profile in BALF [33], while Jang et al. demonstrated that the abundances of *Neisseria* and *Veillonella dispar* differed significantly in relation to PD-L1 expression levels and immunotherapy responses [34]. However, no systemic analysis of the microbiome in BALF of SCC patients has been reported. Our study revealed an association between the bacterial genus Streptococcus and achievement of pCR, but we did not establish a causative relationship. While provocative, further research is required to determine any predictive or functional role of lung microbiome profiles in determining neoadjuvant treatment efficacy.

A recent meta-analysis by Marinelli et al. [35] reviewed published neoadjuvant and preoperative chemo-immunotherapy trials, demonstrating an event-free survival (EFS) advantage in PD-L1 negative tumors. The study showed improved EFS in the experimental arm (hazard ratio, HR 0.55), irrespective of stage, histology, or PD-L1 expression (PD-L1 negative, HR 0.74). Subgroup analysis for PD-L1 did not reveal significant differences in the likelihood of pCR between PD-L1 positive and negative NSCLC patients (*p* = 0.36). The European Medicines Agency (EMA) has limited the use of nivolumab plus chemotherapy to PD-L1-positive tumors. In our study, while no statistically significant difference in PD-L1 expression was observed between pCR and non-pCR groups, there was a trend toward higher PD-L1 TPS in pCR patients. Considering the potential benefits of neoadjuvant chemo-immunotherapy in PD-L1 negative patients, as suggested by the meta-analysis, further research is warranted to explore this approach’s efficacy in this subgroup. Additionally, it is crucial to consider alternative biomarkers beyond PD-L1 expression to guide treatment decisions, ensuring that patients with PD-L1 negative tumors are not excluded from potentially beneficial neoadjuvant therapies.

In light of these findings, we propose that not all SCC patients require the same duration of perioperative therapy, which can last up to 6–12 months. Our study suggests that patients with high TNB score and GEP-18 score, high *IFNG* expression level and M1/M2 macrophages ratio, as well as more CD8 + T cells detected in tumor tissue at baseline, high bacterial genus *Streptococcus* detected in BALF at baseline, or those who achieve ctDNA clearance in peripheral blood after neoadjuvant therapy may be effectively treated with a shorter course of four cycles of perioperative treatment with immunotherapy plus chemotherapy. This personalized approach to treatment may benefit patients with potentially resectable stage IIIA-IIIB(N2) SCC. Nevertheless, further prospective intervention studies are necessary to validate these findings.

This study had several limitations. First, one potential limitation is the staging procedures used for patient selection. International guidelines recommend preoperative staging with PET scan and tissue confirmation with EBUS/EUS for cN1 tumors, tumors ≥ 3 cm, or central tumors [36]. In our study, we utilized clinical radiographic staging, primarily through enhanced CT scans, to assess nodal involvement, reserving PET-CT and invasive mediastinal staging (EBUS/EUS, mediastinoscopy) for cases where CT results were inconclusive. While this approach allowed for a more pragmatic study design, it may have led to some variability in the accuracy of staging across patients. Future studies should consider incorporating PET scan and EBUS/EUS confirmation for all patients meeting the criteria outlined in the guidelines to ensure more precise and consistent staging. Second, the sample size is relatively small, and our study lacks a randomized control group for comparison. Third, the follow-up period for survival data was limited at the time of data cutoff, and longer-term follow-up is necessary to fully evaluate the impact of neoadjuvant therapy on survival outcomes. While several tumor-based biomarkers were associated with achieving pCR in our study, along with the novel finding of an association with Streptococcus abundance, these results remain early and do not supplant pathological response as the primary correlate to survival outcomes after neoadjuvant therapy. Analyses identifying ctDNA clearance, gene expression profiles, tumor mutation/neoantigen burden, and microbiome signatures should be considered hypothesis-generating. Additional prospective validation is necessary before clinical application.

## Conclusions

In conclusion, our study demonstrated that the combination of four cycles of perioperative neoadjuvant treatment with tislelizumab combined with chemotherapy resulted in a promising pCR and MPR rate with acceptable toxicity in patients with potentially resectable stage IIIA-IIIB (N2) SCC. This short-term treatment effectively downstaged a substantial proportion of stage IIIA/IIIB (N2) tumors, with 84.4% of patients achieving pathological T downstaging and 71.9% achieving nodal downstaging after neoadjuvant therapy. These findings suggest that the condensed perioperative chemo-immunotherapy regimen may improve the likelihood of successful surgical resection in this patient population. Furthermore, our study identified several potential predictive biomarkers of pathological response to the combined therapy. We also preliminarily illustrated the potential role of ctDNA dynamics in guiding adjuvant therapy. The pCR group had a significantly higher GEP score than the non-pCR group, and the bacterial genus *Streptococcus* in BALF at baseline was found to be an effective predictor of treatment efficacy. Future studies with larger patient cohorts and randomized control groups are necessary to validate our results.

## Supplementary Information

Additional file 1: Figure S1: TACT trial consort diagram. Description of TACT trial intention-to-treat and full-analysis sets. Figure S2: ctDNA concentration at baseline (A) and ctDNA positive rate after neoadjuvant treatment (B) between pCR and non-pCR groups. (C) EFS of patients with ctDNA clearance and without ctDNA clearance after neoadjuvant. Figure S3: (A) abundance score for each immune cell type in tumor tissue. (B) ROC curve for the prediction of pCR using median TPM expression level of IFNG as cutoff. (C) correlation between IFNG expression level and CD8 + T cell abundance. (D) correlation between IFNG expression level and M1 Macrophages abundance. (E) EFS of patients with high and low expression level of IFNG when using the upper quartile as cutoff. (F) TMB between pCR group and non-pCR group. (G) PD-L1 tumor proportion score between pCR and non-pCR groups.

Additional file 2: Supplementary Table 1. Characterization of all the Participants

## Data Availability

The raw dataset used and analyzed during the current study are available from the corresponding author on reasonable request. And these processed data can be obtained in the supplementary table. C-SA, B-L, and S–S are employees of the Burning Rock Biotech Inc. All other authors declare no competing interests.

## Abbreviations

AEs: Adverse events
BALF: Bronchoalveolar lavage fluid
CTCAE: Common Terminology Criteria for Adverse Events
ctDNA: Circulating tumor DNA
DFS: Disease-free survival
ECOG: Eastern Cooperative Oncology Group
EFS: Event-free survival
GEP: Gene Expression Profile
MPR: Major Pathologic Response
NSCLC: Non-small cell lung cancer
ORR: Overall response rate
pCR: Pathological Complete Response
PD-1: Programmed Cell Death Protein 1
PD-L1: Programmed Cell Death-Ligand 1
SCC: Squamous cell lung carcinoma
TMB: Tumor mutation burden
TNB: Tumor neoantigen burden
WTS: Whole transcriptome sequencing

## References

[1] Remon J, Levy A, Singh P, Hendriks LEL, Aldea M, Arrieta O. Current challenges of unresectable stage III NSCLC: are we ready to break the glass ceiling of the PACIFIC trial? Ther Adv Med Oncol. 2022;14:17588359221113268. 35923929 10.1177/17588359221113268 PMC9340398

[2] Evison M, AstraZeneca UKL. The current treatment landscape in the UK for stage III NSCLC. Br J Cancer. 2020;123(Suppl 1):3–9. 33293670 10.1038/s41416-020-01069-z PMC7735211

[3] Cao C, Le A, Bott M, Yang CJ, Gossot D, Melfi F, et al. Meta-analysis of neoadjuvant immunotherapy for patients with resectable non-small cell lung cancer. Curr Oncol. 2021;28(6):4686–701. 34898553 10.3390/curroncol28060395 PMC8628782

[4] Deboever N, Eisenberg M, Chidi A, Sepesi B. The role of immunotherapy and targeted therapy in the multimodal therapy for resectable lung cancer. J Surg Oncol. 2023;127(2):275–81. 36630093 10.1002/jso.27166

[5] Uprety D, West HJ. Perioperative therapy for resectable non-small-cell lung cancer: weighing options for the present and future. JCO Oncol Pract. 2023;19(7):403–9. 10.1200/OP.23.00014 37023371

[6] Wakelee H, Liberman M, Kato T, Tsuboi M, Lee S-H, Gao S, et al. Perioperative pembrolizumab for early-stage non–small-cell lung cancer. New England J Med. 2023;389(6):491–503. 10.1056/NEJMoa2302983 PMC11074923 37272513

[7] Felip E, Altorki N, Zhou C, Csoszi T, Vynnychenko I, Goloborodko O, et al. Adjuvant atezolizumab after adjuvant chemotherapy in resected stage IB-IIIA non-small-cell lung cancer (IMpower010): a randomised, multicentre, open-label, phase 3 trial. Lancet. 2021;398(10308):1344–57. 34555333 10.1016/S0140-6736(21)02098-5

[8] O’Brien M, Paz-Ares L, Marreaud S, Dafni U, Oselin K, Havel L, et al. Pembrolizumab versus placebo as adjuvant therapy for completely resected stage IB-IIIA non-small-cell lung cancer (PEARLS/KEYNOTE-091): an interim analysis of a randomised, triple-blind, phase 3 trial. Lancet Oncol. 2022;23(10):1274–86. 36108662 10.1016/S1470-2045(22)00518-6

[9] Forde PM, Spicer J, Lu S, Provencio M, Mitsudomi T, Awad MM, et al. Neoadjuvant nivolumab plus chemotherapy in resectable lung cancer. N Engl J Med. 2022;386(21):1973–85. 35403841 10.1056/NEJMoa2202170 PMC9844511

[10] Provencio M, Serna-Blasco R, Nadal E, Insa A, Garcia-Campelo MR, Casal Rubio J, et al. Overall survival and biomarker analysis of neoadjuvant nivolumab plus chemotherapy in operable stage IIIA non-small-cell lung cancer (NADIM phase II trial). J Clin Oncol. 2022;40(25):2924–33. 35576508 10.1200/JCO.21.02660 PMC9426809

[11] Zappa C, Mousa SA. Non-small cell lung cancer: current treatment and future advances. Transl Lung Cancer Res. 2016;5(3):288–300. 27413711 10.21037/tlcr.2016.06.07 PMC4931124

[12] Wang J, Lu S, Yu X, Hu Y, Sun Y, Wang Z, et al. Tislelizumab plus chemotherapy vs chemotherapy alone as first-line treatment for advanced squamous non-small-cell lung cancer: a phase 3 randomized clinical trial. JAMA Oncol. 2021;7(5):709–17. 33792623 10.1001/jamaoncol.2021.0366 PMC8017481

[13] Travis WD, Dacic S, Wistuba I, Sholl L, Adusumilli P, Bubendorf L, et al. IASLC multidisciplinary recommendations for pathologic assessment of lung cancer resection specimens after neoadjuvant therapy. J Thorac Oncol. 2020;15(5):709–40. 32004713 10.1016/j.jtho.2020.01.005 PMC8173999

[14] Chen K, Shen H, Wu S, Zhu P, Wang C, Lizaso A, et al. Abstract 5916: Tumor-informed patient-specific panel outperforms tumor-naïve and tumor-informed fixed panel for circulating tumor DNA (ctDNA)-based postoperative monitoring of non-small cell lung cancer (NSCLC). Cancer Res. 2022;82(12_Supplement):5916.

[15] Provencio M, Nadal E, Insa A, Garcia-Campelo MR, Casal-Rubio J, Domine M, et al. Neoadjuvant chemotherapy and nivolumab in resectable non-small-cell lung cancer (NADIM): an open-label, multicentre, single-arm, phase 2 trial. Lancet Oncol. 2020;21(11):1413–22. 32979984 10.1016/S1470-2045(20)30453-8

[16] Provencio-Pulla M, Nadal E, Larriba JLG, Martinez-Marti A, Bernabé R, Bosch-Barrera J, et al. Nivolumab + chemotherapy versus chemotherapy as neoadjuvant treatment for resectable stage IIIA NSCLC: Primary endpoint results of pathological complete response (pCR) from phase II NADIM II trial. J Clin Oncol. 2022;40(16_suppl):8501.

[17] Yan W, Zhong WZ, Liu YH, Chen Q, Xing W, Zhang Q, et al. Adebrelimab (SHR-1316) in combination with chemotherapy as perioperative treatment in patients with resectable stage II to III NSCLCs: an open-label, multicenter, phase 1b trial. J Thorac Oncol. 2023;18(2):194–203. 36191882 10.1016/j.jtho.2022.09.222

[18] Heymach JV HD, Mitsudomi T, et al., editor AEGEAN: a phase 3 trial of neoadjuvant durvalumab + chemotherapy followed by adjuvant durvalumab in patients with resectable NSCLC. 2023 AACR Annual Meeting; 2023 April 14–19; Orlando. United States.

[19] Wu L, Zhang W, Zhang P, Wang W, Fang W, Xing W, et al. Perioperative toripalimab + platinum-doublet chemotherapy vs chemotherapy in resectable stage II/III non-small cell lung cancer (NSCLC): Interim event-free survival (EFS) analysis of the phase III Neotorch study. J Clin Oncol. 2023;41(36_suppl).

[20] Wakelee H, Liberman M, Kato T, Tsuboi M, Lee SH, Gao S, et al. Perioperative Pembrolizumab for Early-Stage Non-Small-Cell Lung Cancer. N Engl J Med. 2023. 10.1056/NEJMoa2302983 PMC11074923 37272513

[21] Qiu B, Guo W, Zhang F, Lv F, Ji Y, Peng Y, et al. Dynamic recurrence risk and adjuvant chemotherapy benefit prediction by ctDNA in resected NSCLC. Nat Commun. 2021;12(1):6770. 34799585 10.1038/s41467-021-27022-z PMC8605017

[22] Xia L, Mei J, Kang R, Deng S, Chen Y, Yang Y, et al. Perioperative ctDNA-based molecular residual disease detection for non-small cell lung cancer: a prospective multicenter cohort study (LUNGCA-1). Clin Cancer Res. 2022;28(15):3308–17. 34844976 10.1158/1078-0432.CCR-21-3044

[23] Zhang JT, Liu SY, Gao W, Liu SM, Yan HH, Ji L, et al. Longitudinal undetectable molecular residual disease defines potentially cured population in localized non-small cell lung cancer. Cancer Discov. 2022;12(7):1690–701. 35543554 10.1158/2159-8290.CD-21-1486 PMC9394392

[24] Zhou C, Das Thakur M, Srivastava MK, Zou W, Xu H, Ballinger M, et al. 2O IMpower010: Biomarkers of disease-free survival (DFS) in a phase III study of atezolizumab (atezo) vs best supportive care (BSC) after adjuvant chemotherapy in stage IB-IIIA NSCLC. Ann Oncol. 2021;32:S1374.

[25] Yue D, Liu W, Chen C, Zhang T, Ma Y, Cui L, et al. Circulating tumor DNA predicts neoadjuvant immunotherapy efficacy and recurrence-free survival in surgical non-small cell lung cancer patients. Transl Lung Cancer Res. 2022;11(2):263–76. 35280315 10.21037/tlcr-22-106 PMC8902085

[26] Wang P, Chen Y, Wang C. Beyond tumor mutation burden: tumor neoantigen burden as a biomarker for immunotherapy and other types of therapy. Front Oncol. 2021;11:672677. 33996601 10.3389/fonc.2021.672677 PMC8117238

[27] Boutilier AJ, Elsawa SF. Macrophage polarization states in the tumor microenvironment. Int J Mol Sci. 2021;22(13):6995. 10.3390/ijms22136995 PMC8268869 34209703

[28] Ma J, Liu L, Che G, Yu N, Dai F, You Z. The M1 form of tumor-associated macrophages in non-small cell lung cancer is positively associated with survival time. BMC Cancer. 2010;10:112. 20338029 10.1186/1471-2407-10-112 PMC2851690

[29] Casarrubios M, Provencio M, Nadal E, Insa A, Del Rosario Garcia-Campelo M, Lazaro-Quintela M, et al. Tumor microenvironment gene expression profiles associated to complete pathological response and disease progression in resectable NSCLC patients treated with neoadjuvant chemoimmunotherapy. J Immunother Cancer. 2022;10(9):e005320. 10.1136/jitc-2022-005320 PMC9528578 36171009

[30] Schroder K, Hertzog PJ, Ravasi T, Hume DA. Interferon-gamma: an overview of signals, mechanisms and functions. J Leukoc Biol. 2004;75(2):163–89. 14525967 10.1189/jlb.0603252

[31] Liu X, Cheng Y, Zang D, Zhang M, Li X, Liu D, et al. The role of gut microbiota in lung cancer: from carcinogenesis to immunotherapy. Front Oncol. 2021;11:720842. 34490119 10.3389/fonc.2021.720842 PMC8417127

[32] Khan MAW, Ologun G, Arora R, McQuade JL, Wargo JA. Gut microbiome modulates response to cancer immunotherapy. Dig Dis Sci. 2020;65(3):885–96. 32067144 10.1007/s10620-020-06111-x PMC7678709

[33] Masuhiro K, Tamiya M, Fujimoto K, Koyama S, Naito Y, Osa A, et al. Bronchoalveolar lavage fluid reveals factors contributing to the efficacy of PD-1 blockade in lung cancer. JCI Insight. 2022;7(9):e157915. 10.1172/jci.insight.157915 PMC9090256 35389889

[34] Grenda A, Iwan E, Chmielewska I, Krawczyk P, Giza A, Bomba A, et al. Presence of Akkermansiaceae in gut microbiome and immunotherapy effectiveness in patients with advanced non-small cell lung cancer. AMB Express. 2022;12(1):86. 35792976 10.1186/s13568-022-01428-4 PMC9259768

[35] Marinelli D, Gallina FT, Pannunzio S, Di Civita MA, Torchia A, Giusti R, et al. Surgical and survival outcomes with perioperative or neoadjuvant immune-checkpoint inhibitors combined with platinum-based chemotherapy in resectable NSCLC: A systematic review and meta-analysis of randomised clinical trials. Crit Rev Oncol Hematol. 2023;192:104190. 37871779 10.1016/j.critrevonc.2023.104190

[36] De Leyn P, Dooms C, Kuzdzal J, Lardinois D, Passlick B, Rami-Porta R, et al. Revised ESTS guidelines for preoperative mediastinal lymph node staging for non-small-cell lung cancer. Eur J Cardiothorac Surg. 2014;45(5):787–98. 24578407 10.1093/ejcts/ezu028

