# Supplementary material for: Optimizing perioperative treatment for potentially resectable stage III squamous cell lung carcinoma: promising results of a condensed four-cycle regimen with tislelizumaband chemotherapy
Source: BMC Med. 2024 Jun 10;22:234. doi: 10.1186/s12916-024-03462-4 (PMC11163755; doi:10.1186/s12916-024-03462-4)
Supplement: Supplementary file 1 — Additional file 1: Figure S1: TACT trial consort diagram. Description of TACT trial intention-to-treat and full-analysis sets. Figure S2: ctDNA concentration at baseline (A) and ctDNA positive rate after neoadjuvant treatment (B) between pCR and non-pCR groups. (C) EFS of patients with ctDNA clearance and without ctDNA clearance after neoadjuvant. Figure S3: (A) abundance score for each immune cell type in tumor tissue. (B) ROC curve for the prediction of pCR using median TPM expression level of IFNG as cutoff. (C) correlation between IFNG expression level and CD8 + T cell abundance. (D) correlation between IFNG expression level and M1 Macrophages abundance. (E) EFS of patients with high and low expression level of IFNG when using the upper quartile as cutoff. (F) TMB between pCR group and non-pCR group. (G) PD-L1 tumor proportion score between pCR and non-pCR groups. [file 12916_2024_3462_MOESM1_ESM.docx]

**Supplementary Materials for Optimizing Perioperative Treatment for Potentially Resectable Stage III Squamous Cell Lung Carcinoma: Promising Results of a Condensed Four-Cycle Regimen with Tislelizumab and Chemotherapy**

Jianzhen Shan*^,1,2, #,^ Zhen Liu^1,2, #^, Songan Chen^3^, Chengli Du^4^, Bing Li^3^, Lingxiang Ruan^5^, Mei Kong^6^, Lingjie Wang^1,2^, Miaoyan Du^1,2^, Shuo Shi^7^, Guoliang Qiao^1,2^, Tian Tian^1,2^, Zhengliang Tu*^,4^

**Supplementary methods**

**PD-L1 expression evaluation**

Tumor biopsies used for PD-L1 expression evaluation were obtained before treatment. PD-L1 positive was defined as a tumor proportion score (TPS) of ≥1% by PD-L1 IHC 22C3 pharmDx (Dako, Agilent Technologies, Santa Clara, CA, USA). Cases without enough or eligible tumor specimens for scoring were defined as not applicable (NA).

**Whole exome sequencing and data analysis**

The whole-exome sequencing (WES) library prep was performed using Twist Human Core Exome kit (Twist Bioscience, South San Francisco, CA, US) according to the manufacturer's recommendations. Briefly, genomic DNA was extracted from formalin-fixed and parrffin-embedded (FFPE) tumor tissues and paired white blood cell (WBC) and then were sheared using M220 Focused-ultrasonicator (Covaris, Woburn, MA, US) to peak size 200 bp. Sheared DNA samples were end-repaired and dA-tailed, followed by ligation with Universal Adapters. After post-ligation cleanup, ligated products were polymerase chain reaction (PCR) amplified with index primers. The number of amplification cycles used was varied during library preparation according to the manufacturer's recommendations. Exome capture was performed with Twist Fast Hybridization and Wash Kit, using a 33Mb Human Core Exome panel and a customized-designed supplementary panel. Up to 8 libraries were multiplexed in one capture reaction, and 400 ng of each library was used as input. Final libraries of WES were quantified using Qubit™ dsDNA HS assay (Thermo Fisher, Carlsbad, CA, US). After library size-distribution determination by using a LabChip GX Touch System, the libraries were sequenced on NovaSeq 6000 sequencer (Illumina, San Diego, CA, US) with 2×151bp pair-end reads with unique dual index, with a mean target coverage of 500x for tumor samples and 150x for paired normal samples.

All the analyses on WES reads data were performed using Illumina DRAGEN Bio-IT Platform (Illumina, Inc., San Diego, CA, US) unless otherwise indicated. FastQ files were generated from raw BCL data using the DRAGEN Bcl Convert pipeline v3.7.4. Adapters were trimmed by using fastp v0.23.0^1^, and reads length <50 bp were discarded. Clean reads were mapped to the human reference genome (NCBI GRCh37; hg19)^2^, and PCR duplicates were marked for filtering in the downstream analysis. As a quality-control (QC) process for all samples captured by the Human Core Exome panel, tumor and paired normal alignments were checked for multiple QC parameters using the in-house software to assess capture efficiency, coverage uniformity, and library complexity. Single nucleotide variants (SNVs) and insertions/deletions (INDELs) were called if the variant supporting reads≥5 and mutation VAF≥3%. According to ExAC, 1000 Genomes, dbSNP, and ESP6500SI-V2 databases, variants with a population frequency>0.5% in tumors were grouped as single-nucleotide polymorphisms (SNPs) and excluded from further analysis. In order to get real somatic mutations, for each variant, the ratio of tumor AF to normal AF was calculated. All variants with the ratio less than 3 or both AFs greater than 10% were excluded. All variants passing the applied filters were annotated with ANNOVAR^3^ and SnpEff v3.6^4^.

Tumor mutational burden (TMB) per patient was computed as a ratio between the total number of nonsynonymous mutations detected and the total coding region size of the panel. Tumor neoantigen burden (TNB) score was calculated as the total number of all mutations which may generate neoantigens per megabase.

**Whole transcriptome sequencing and data analysis**

RNA was isolated from FFPE samples using an MagPure FFPE DNA/RNA LQ Kit (Magen Biotechnology, Guangzhou, China). Qubit RNA HS (Thermor Fisher) was used to quantify the total amount and evaluate the purity of extracted RNA. 2100 RNA Nano/Pico kit (Agilent) was used for assessing RNA integrity. The RNA template strand was reverse-transcribed into cDNA, followed by second strand synthesis and cDNA end repair by adding a dUTP adapter. Subsequently, sequencing adapters were added at both ends of the DNA followed by non-capture PCR amplification. The amplified libraries were sequenced on novaseq 6000 (Illumina) with a target data of 6G (40Million X150bp) per sample. The raw reads were trimmed with trimmomatic (v0.36) and trimmed reads was processed with unique molecular identifiers (UMIs) information by in-house scripts to generate consensus reads^5^ . Then the consensus reads were aligned against the human reference genome (hg19) by STAR (v2.7.3a)^6^. Gene quantification were performed with HTSeq count (v0.12.4) in union mode^7^. Transcript level quantification (TPM) was performed using Salmon (v1.2.1)^8^.

The abundance score for each immune cell type was calculated by estimating relative subsets of RNA transcripts using CIBERSORT under absolute mode^9^. The gene expression profile (GEP) analysis included 18 T-cell-inflamed genes related to antigen presentation, chemokine expression, cytolytic activity, and adaptive immune resistance, including CCL5, CD27, CD274 (PD-L1), CD276 (B7-H3), CD8A, CMKLR1, CXCL9, CXCR6, HLA-DQA1, HLA-DRB1, HLA-E, IDO1, LAG3, NKG7, PDCD1LG2 (PDL2), PSMB10, STAT1, and TIGTT. The GEP score was computed as the weighted sum of the housekeeping normalized values of the 18 genes for a given sample as described previously^10, 11^.

**Circulating tumor DNA (ctDNA) analysis**

ctDNA analysis was performed with the use of a tumor-informed personalized ctDNA panel for whole-exome sequencing (PROPHET, Burning Rock Biotech, Guangzhou, China). Briefly, patient-specific somatic variants were identified by analysis of primary tumor and matched normal white blood cell WES samples. For a given set of variants, up to 50 highly ranked variants with a VAF≥3.0% were selected for panel design. The biotinylated capture probe pool was produced in-house based on each personalized panel design. The library preparation and enrichment process were performed using Burning Rock HS unique molecular identifier (UMI) library preparation kit. Ultra-deep UMI-based sequencing was performed on a NovaSeq 6000 platform (Illumina, San Diego, CA, US), with 2×151bp paired-end reads and a target raw depth of 100,000x. Clearance of ctDNA was defined as detectable levels of ctDNA before Neoadjuvant Treatment to undetectable ctDNA after neoadjuvant treatment. Absolute ctDNA levels were expressed as mean tumor molecules per milliliter (MTM/ml) of plasma, based on variant allele frequencies and quantity of cell free DNA (cfDNA)

**Microbial Sequencing of bronchoalveolar lavage fluid (BALF) supernatant**

Prior to neoadjuvant treatment, bronchoscopy was performed on the patients, during which a suitable volume of normal saline was introduced to the lesion site to collect BALF. Microbial DNA was then extracted from each BALF sample using the PowerSoil DNA Isolation Kit (QIAGEN). Specific primers were utilized to amplify target genes, including 16S rRNA, 18S rRNA, and ITS genes from various regions, such as 16SV4, 16SV3, 16SV3-V4, 16SV4-V5, 18SV4, 18SV9, ITS1, ITS2, and ArcV4. DNA libraries were prepared, and the NovaSeq PE250 high-throughput sequencing platform was chosen for this study. Raw sequencing data were processed and analyzed, involving the removal of low-quality reads, filtering of host sequences, assembly and annotation of the sequences, and functional analysis. The results were statistically interpreted and analyzed for further investigation.

**Supplementary figures**


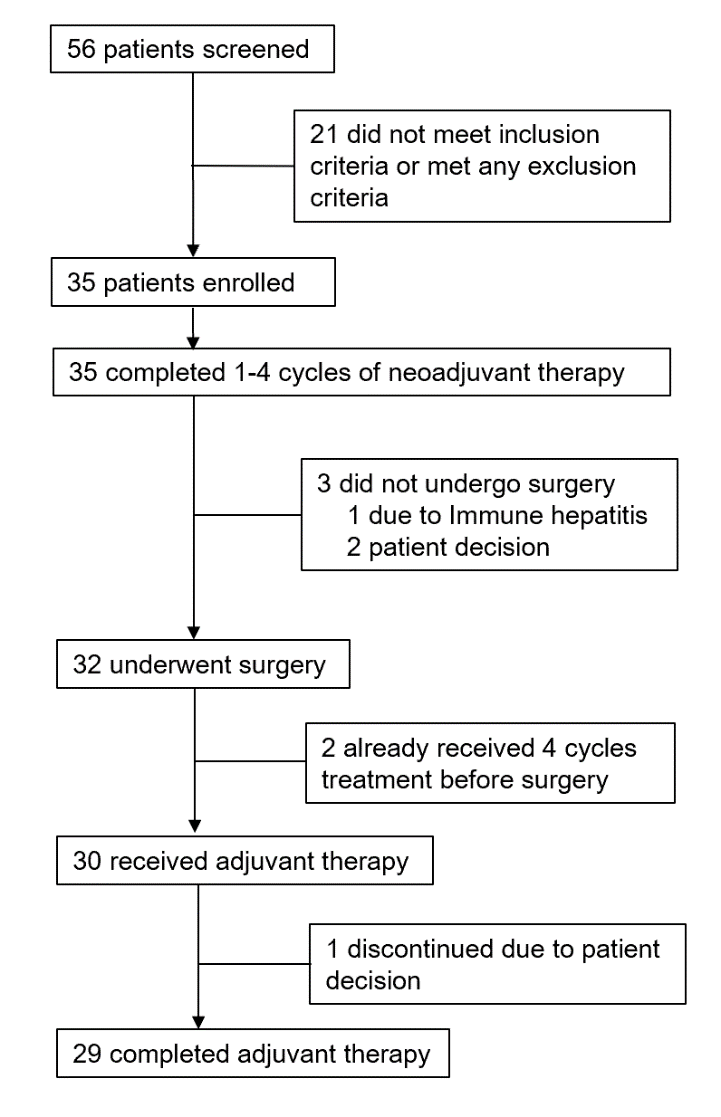


**Figure S1**. TACT trial consort diagram. Description of TACT trial intention-to-treat and full-analysis sets.


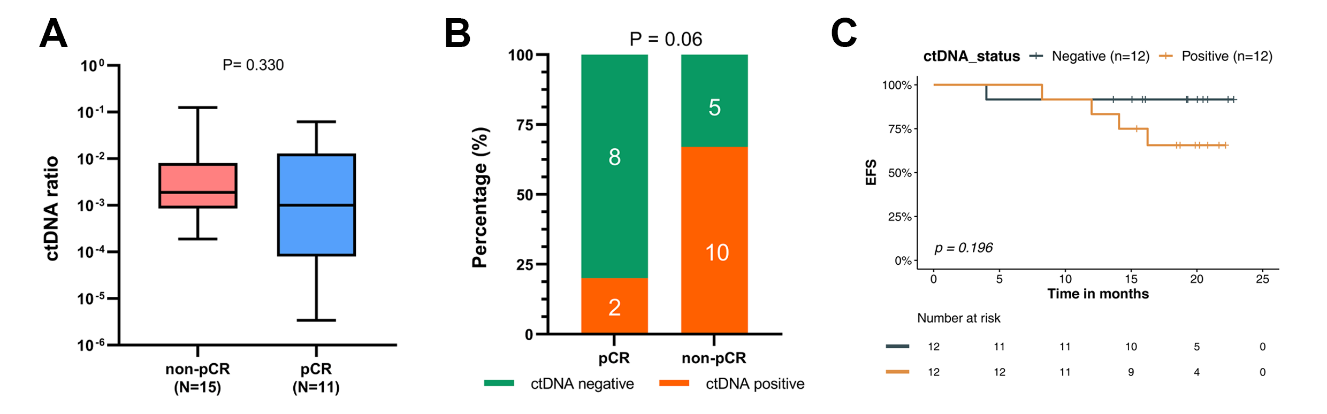


**Figure S2**. ctDNA concentration at baseline (A) and ctDNA positive rate after neoadjuvant treatment (B) between pCR and non-pCR groups. (C) EFS of patients with ctDNA clearance and without ctDNA clearance after neoadjuvant.


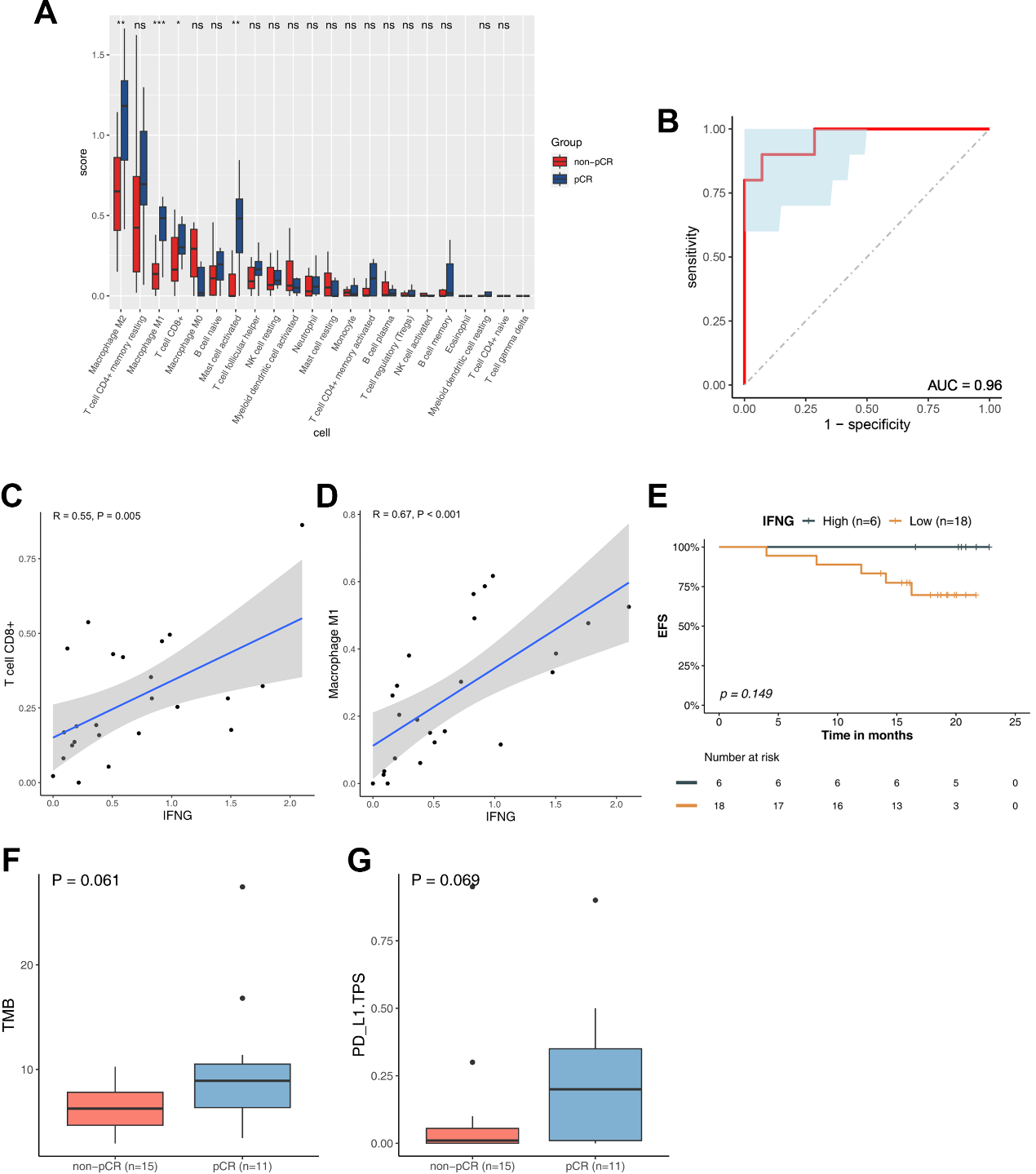


**Figure S3**. (A) abundance score for each immune cell type in tumor tissue. (B) ROC curve for the prediction of pCR using median TPM expression level of IFNG as cut-off. (C) correlation between IFNG expression level and CD8+T cell abundance. (D) correlation between IFNG expression level and M1 Macrophages abundance. (E) EFS of patients with high and low expression level of IFNG when using the upper quartile as cut-off. (F) TMB between pCR group and non-pCR group. (G) PD-L1 tumor proportion score between pCR and non-pCR groups.

**References**

1. Chen S, Zhou Y, Chen Y, et al. fastp: an ultra-fast all-in-one FASTQ preprocessor. *Bioinformatics* 2018;34:i884-i890.

2. Lander ES, Linton LM, Birren B, et al. Initial sequencing and analysis of the human genome. *Nature* 2001;409:860-921.

3. Wang K, Li M, Hakonarson H. ANNOVAR: functional annotation of genetic variants from high-throughput sequencing data. *Nucleic Acids Res* 2010;38:e164.

4. Cingolani P, Platts A, Wang le L, et al. A program for annotating and predicting the effects of single nucleotide polymorphisms, SnpEff: SNPs in the genome of Drosophila melanogaster strain w1118; iso-2; iso-3. *Fly (Austin)* 2012;6:80-92.

5. Bolger AM, Lohse M, Usadel B. Trimmomatic: a flexible trimmer for Illumina sequence data. *Bioinformatics* 2014;30:2114-2120.

6. Dobin A, Davis CA, Schlesinger F, et al. STAR: ultrafast universal RNA-seq aligner. *Bioinformatics* 2013;29:15-21.

7. Anders S, Pyl PT, Huber W. HTSeq--a Python framework to work with high-throughput sequencing data. *Bioinformatics* 2015;31:166-169.

8. Patro R, Duggal G, Love MI, et al. Salmon provides fast and bias-aware quantification of transcript expression. *Nat Methods* 2017;14:417-419.

9. Chen B, Khodadoust MS, Liu CL, et al. Profiling Tumor Infiltrating Immune Cells with CIBERSORT. *Methods Mol Biol* 2018;1711:243-259.

10. Ott PA, Bang YJ, Piha-Paul SA, et al. T-Cell-Inflamed Gene-Expression Profile, Programmed Death Ligand 1 Expression, and Tumor Mutational Burden Predict Efficacy in Patients Treated With Pembrolizumab Across 20 Cancers: KEYNOTE-028. *J Clin Oncol* 2019;37:318-327.

11. Ayers M, Lunceford J, Nebozhyn M, et al. IFN-gamma-related mRNA profile predicts clinical response to PD-1 blockade. *J Clin Invest* 2017;127:2930-2940.
